# Diet modulates the relationship between immune gene expression and functional immune responses

**DOI:** 10.1016/j.ibmb.2019.04.009

**Published:** 2019-06

**Authors:** Sheena C. Cotter, Catherine E. Reavey, Yamini Tummala, Joanna L. Randall, Robert Holdbrook, Fleur Ponton, Stephen J. Simpson, Judith A. Smith, Kenneth Wilson

**Affiliations:** a School of Life Sciences, University of Lincoln, Brayford Pool, Lincoln, LN6 7TS, UK; b Lancaster Environment Centre, Lancaster University, Lancaster, LA1 4YQ, UK; c Charles Perkins Centre, University of Sydney, NSW, 2006, Australia; d Department of Biological Sciences, Macquarie University, NSW, 2109, Australia; e School of Forensic and Applied Sciences, University of Central Lancashire, Preston, Lancashire, PR1 2HE, UK

**Keywords:** Nutritional ecology, Host-pathogen interaction, Immunity, *Spodoptera*, *Xenorhabdus*, Diet, Bacteria, Resistance, Tolerance, Insect, Geometric framework

## Abstract

Nutrition is vital to health and the availability of resources has long been acknowledged as a key factor in the ability to fight off parasites, as investing in the immune system is costly. Resources have typically been considered as something of a “black box”, with the quantity of available food being used as a proxy for resource limitation. However, food is a complex mixture of macro- and micronutrients, the precise balance of which determines an animal's fitness. Here we use a state-space modelling approach, the Geometric Framework for Nutrition (GFN), to assess for the first time, how the balance and amount of nutrients affects an animal's ability to mount an immune response to a pathogenic infection.

*Spodoptera littoralis* caterpillars were assigned to one of 20 diets that varied in the ratio of macronutrients (protein and carbohydrate) and their calorie content to cover a large region of nutrient space. Caterpillars were then handled or injected with either live or dead *Xenorhabdus nematophila* bacterial cells. The expression of nine genes (5 immune, 4 non-immune) was measured 20 h post immune challenge. For two of the immune genes (PPO and Lysozyme) we also measured the relevant functional immune response in the hemolymph. Gene expression and functional immune responses were then mapped against nutritional intake.

The expression of all immune genes was up-regulated by injection with dead bacteria, but only those in the IMD pathway (Moricin and Relish) were substantially up-regulated by both dead and live bacterial challenge. Functional immune responses increased with the protein content of the diet but the expression of immune genes was much less predictable.

Our results indicate that diet does play an important role in the ability of an animal to mount an adequate immune response, with the availability of protein being the most important predictor of the functional (physiological) immune response. Importantly, however, immune gene expression responds quite differently to functional immunity and we would caution against using gene expression as a proxy for immune investment, as it is unlikely to be reliable indicator of the immune response, except under specific dietary conditions.

## Highlights

• High protein diets improved survival after live bacterial infection.
• Injection with dead bacteria increased expression of Toll and IMD immune genes.
• Injection with live bacteria inhibited immune gene expression (GE).
• The ratio and concentration of macronutrients in the diet affected GE.
• GE only predicted functional immune activity at high levels of dietary protein.

## Graphical abstract

**Figure:**
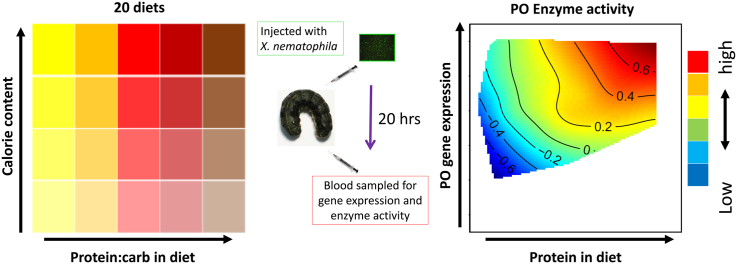

## 1 Introduction

It has long been recognised the role that “good nutrition” plays in human health, with both under-nutrition and obesity resulting in disease (Mokdad et al., 2001; Muller and Krawinkel, 2005; Samartin and Chandra, 2001). Poor nutrition can also impact the response to parasites, with evidence for both energy and protein deficits reducing the ability to fight infection (Kuvibidila et al., 1993; Field et al., 2002; Cunningham-Rundles et al., 2005). Studies have shown that starvation can compromise immune capability across a broad range of host taxa. For example, laboratory mice were found to have fewer T cells in the spleen and thymus during starvation, with numbers recovering once feeding was reinstated (Wing et al., 1988). Furthermore, injection with *Listeria monocytogenes* during starvation reduced the ability of the mice to develop antibodies against this bacterium (Wing et al., 1988). Food restriction, rather than starvation can have similar effects. Food-restricted Yellow-legged gulls, *Larus cachinnans*, were found to have reduced cell-mediated immunity (Alonso-Alvarez and Tella, 2001) and mice on a long-term calorie-restricted diet were found to die more rapidly from sepsis after gut puncture than those fed *ad libitum* (Alonso-Alvarez and Tella, 2001). Comparable responses have been shown in invertebrates; bumble bees died more rapidly during starvation if their immune systems were stimulated by artificial parasites, suggesting that mounting an immune response is energetically costly (Moret and Schmid-Hempel, 2000). Similarly, starved bumble bees were more likely to die from a gut parasite, *Crithida bombi,* than hosts with adequate nutrition (Brown et al., 2000).

However, nutrition is much more complex than simply a source of energy, being a vital mixture of macro- (carbohydrates, fats and proteins) and micro-nutrients (vitamins and minerals), the amount and balance of which determine an animal's fitness (Simpson et al., 2004). Several studies have examined how shifting the balance of macronutrients in the diet affects immune responses and the outcome of infection, without restricting the availability of calories (Graham et al., 2014; Lee et al., 2006; Ponton et al., 2011; Povey et al., 2009, 2014). For example, caterpillars of the armyworms, *Spodoptera littoralis* and *Spodoptera exempta*, show improved immune responses and markedly higher survival after viral infection (Lee et al., 2006; Povey et al., 2014) and bacterial infection (Povey et al., 2009) when their diet is heavily protein-biased. Furthermore, when given the opportunity, infected caterpillars will “self-medicate” with protein, significantly improving their chances of survival (Lee et al., 2006; Povey et al., 2009, 2014).

The studies above strongly suggest that it is the source of the energy in the diet that is key to the response to parasites, rather than the availability of energy *per se*. However, neither food restriction, nor the manipulation of macronutrient balance alone can determine the relative importance of either on host-parasite interactions. To address properly the role of nutrient availability on immunity, both the balance of nutrients in the diet and their quantity need to be simultaneously manipulated. The Geometric Framework for Nutrition (GFN) is a state-space model that allows the association of particular nutrient intakes with outcomes of interest (Simpson and Raubenheimer, 1995), for example, immunity (Ponton et al., 2011, 2013). With the GFN, animals are restricted to diets in which both the balance and availability of nutrients are manipulated, forcing intakes over a wide region of nutrient space, encompassing both over- and under-nutrition, and thereby allowing the additive and interactive effects of specific nutrients on traits of interest to be modelled (Simpson and Raubenheimer, 1995).

The GFN approach has highlighted that the fundamental life-history trade-off between fecundity and longevity is mediated by nutrients across taxa, with longevity generally peaking at low-protein, high-carbohydrate ratios, whilst fecundity tends to peak at much higher relative protein intakes; as such, no diet can maximize both traits (*Drosophila*: (Lee et al., 2008; Jensen et al., 2015); Field crickets: (Maklakov et al., 2008); Queensland Fruit fly: (Fanson et al., 2009); Mice (Solon-Biet et al., 2015)). Similarly, using the GFN, it was found that different immune responses peak in different regions of nutrient space, thereby indicating a nutrient-mediated trade-off within the immune system, and, as for fecundity and longevity, no single diet could maximize multiple immune responses (Cotter et al, 2011). In a recent study, mice were restricted to one of 25 diets varying in their ratio of proteins, fats and carbohydrates and energy density, and their innate immune capacity was measured. It was shown that the balance of T cells indicative of healthy ageing was achieved on a low protein:NPE diet (non-protein energy i.e. carbohydrate plus fat), irrespective of calorie intake (Le Couteur et al., 2015). However, this powerful approach has not yet been taken to assess an animal's immune response to a pathogenic challenge.

Insects have a comparatively simple yet effective immune system that has numerous parallels to the innate immune response of vertebrates (Vilmos and Kurucz, 1998; Leulier and Lemaitre, 2008; Wiesner and Vilcinskas, 2010). It comprises cellular and humoral components that work together to fight invading pathogens. Hemocytes show phagocytic activity against microparasites, much like vertebrate macrophages, and can respond to macroparasites by forming a multi-layered envelope around the invader, in a process called encapsulation, which is subsequently melanised via the action of the phenoloxidase (PO) enzyme (Gupta, 1991). Phenoloxidase is stored in hemocytes in the form of an inactive precursor, Pro-phenoloxidase (PPO), which is activated upon detection of non-self (Gonzalez-Santoyo and Cordoba-Aguilar, 2012). This recognition occurs via the detection of pathogen-associated molecular patterns (PAMPs) such as the peptidoglycan or the lipopolysaccharide components of fungal and bacterial cell walls. Detection stimulates either the *Toll* (fungi and gram-positive bacteria) or IMD pathways (Gram-negative bacteria), via host *pattern recognition receptors* (PRRs) that result in the bespoke production of antimicrobial peptides and the upregulation of constitutive lysozymes, which form the humoral component of the response (Ligoxygakis, 2013; Wiesner and Vilcinskas, 2010).

The strength of the immune response can be measured using standard functional assays of antimicrobial activity or PPO activity in the hemolymph, and the strength of the encapsulation response or phagocytosis can be measured against synthetic parasites injected into the haemocoel (see (Wilson and Cotter, 2013) and references therein). These functional responses have been shown to be indicative of the ability of the animal to fight off parasites (e.g. Lee et al., 2006; Paskewitz and Riehle, 1994; Povey et al., 2009) and so are arguably meaningful measures of immune investment. However, gene expression is also often used as a proxy for investment in specific traits, e.g. immunity (Freitak et al., 2007; Jackson et al., 2011; Woestmann et al., 2017), but few of these studies consider how well the expression of the gene of interest predicts the functional response under the conditions in which they are tested.

There has been a great deal of research examining how well gene transcripts relate to protein abundance across individual genes, but with contradictory findings (Liu et al., 2016). This is not surprising as there are numerous steps between gene expression and the production of the protein, all of which can change the relationship between the two. In cell culture, under steady-state conditions, mRNA transcripts correlate well with protein abundance, typically explaining between 40 and 80% of the variation (Edfors et al., 2016; Jovanovic et al., 2015; Liu et al., 2016). However, multiple factors can affect this relationship. Upregulation of gene expression in response to a perturbation is expected to change the abundance of proteins concordantly, but there can be a delay in this process, such that there is a time lag between mRNA levels and protein abundance, the length of which may differ between genes (Gedeon and Bokes, 2012; Jovanovic et al., 2015). Some genes are constitutively transcribed and translation of the protein occurs only when the correct conditions are met, known as “translation on demand” (Hinnebusch and Natarajan, 2002), meaning that there is no correlation between mRNA and protein levels most of the time. The majority of ecological studies consider gene expression in whole animals, which are hugely more variable than cell cultures, and so we can expect the relationship between gene expression and protein abundance to be further weakened in natural systems. One aspect of variation in whole animals is the availability of resources. Protein production is costly, consuming ∼50% of the ATP in growing yeast cells (Warner, 1999), so we can expect the availability of energy and amino acids to affect the speed and efficacy of translation (Liu et al., 2016). This means that the relationship between the expression of a gene and its protein is likely to change with the resource levels of the animal. To our knowledge, there are no studies comparing how the mRNA-protein relationship changes across nutrient space.

Here we address this gap using a model insect, *Spodoptera littoralis*, (Lepidoptera: Noctuidae), a generalist herbivore. We take a GFN approach, restricting caterpillars to diets that vary in their P:C ratio and energy content, thereby covering a large region of nutrient space. We then challenge the immune system by injecting caterpillars with live or dead bacteria, and measure the expression of 9 genes (5 immune, 4 non-immune), and 3 functional immune responses, which are transcribed by two of the immune genes (PPO and lysozyme) in the hemolymph, thus allowing us to associate gene expression and functional immune responses to nutrient intake, and importantly, to assess how well gene expression predicts the immune response specifically associated with those genes under different dietary conditions.

## 2 Material and methods

### 2.1 Host and pathogen cultures

The *Spodoptera littoralis* culture was established from eggs collected near Alexandria in Egypt in 2011. The colony was reared using single pair matings with around 150 pairs established each generation. Following mating of unrelated adult moths; eggs were laid within 2 days with larvae hatching after a further 3 days. *Spodoptera littoralis* spend approximately 2 weeks in the larval stage, about 7 days of which are spent in the 5th and 6th instars. Larvae were reared individually from the 2nd instar on a semi-artificial wheat germ-based diet (Reeson et al., 1998) in 25 ml polypots until the final larval instar (6th), at which point they were used in the experiments as described below. Insects were maintained at 27°C under a 12:12 light: dark photo regime.

Bacteria were originally supplied by the laboratory of Givaudan and colleagues (Montpellier University, France; *Xenorhabdus nematophila* F1D3 GFP labelled, see (Sicard et al., 2004)). Pure *X. nematophila* F1D3 stocks were stored at −20°C in Eppendorf tubes (500 μl of *X. nematophila* F1D3 in nutrient broth with 500 μl of glycerol). Vortexing ensured that all *X. nematophila* F1D3 cells were coated in glycerol. To revive the stocks for use, 100 μl was added to 10 ml nutrient broth, and incubated at 28°C for up to 48 h (generally stocks had grown sufficiently after 24 h). On the day of experimental bacterial challenge, a sub-culture of the stock was carried out, with 1 ml of the original stock added to 10 ml of nutrient broth and placed in a shaker-incubator for approximately 4 h. This ensured that the bacteria were in log phase prior to challenge. Following the sub-culture, a 1 ml sample was checked for purity under the microscope by looking for non-fluorescent cells, which would indicate contamination. The clean sample was then used to produce a serial dilution in nutrient broth from which the total cell count was determined with fluorescence microscopy, using a haemocytometer with improved Neubauer ruling. The remaining culture was diluted with nutrient broth to the appropriate concentration required for the bacterial challenge. The heat-killed treatment group was established by autoclaving the bacteria for 30 min at 121°C.

### 2.2 Diet manipulation

The aim of the experiments was to tease apart the importance of relative and absolute nutrient effects on immune gene expression and immune protein activity. Therefore, larvae were fed on one of 20 chemically-defined diets that varied in both the protein to carbohydrate (P:C) ratio and calorie density based on (Simpson and Abisgold, 1985) (Table 1). This comprised five P:C ratios (5:1, 2:1, 1:1, 1:2, 1:5) and four calorie densities (326, 612, 756 and 1112 kJ/100 g diet; the remainder of the diet comprising indigestible cellulose (See Table S1 for information about the specific ingredients for each diet). Thus, the 20 diets could be described with respect to the absolute amount of proteins or carbohydrates, by their sum (calorie density), by their ratio (P:C) or by their interaction (P*C). In addition, the absolute amounts of food eaten by the larvae on each diet were recorded so the absolute amount of protein or carbohydrate eaten as opposed to amounts offered could also be used. We were therefore able to define 30 alternative models for describing the relationship between the trait of interest (e.g. Toll expression), and host diet (Table 1). These were then compared using an information theoretic approach by comparing *AIC*_*c*_ values and other model metrics (Burnham and Anderson, 2003; Whittingham et al., 2006).

**Table 1:** Nutritional composition of the 20 chemically-defined diets.

Diet	Protein	Carbs	Fats	Cellulose	Micro-nutrients	Energy	Ratio	P:C
	(g/100 g diet)	(g/100 g diet)	(g/100 g diet)	(g/100 g diet)	(g/100 g diet)	(kJ/100 g diet)	(%)	
**1**	10.5	52.5	1.1	33.0	3.0	1112	0.17	1:5
**2**	7.0	35.0	1.1	54.0	3.0	756	0.17	1:5
**3**	5.6	28.0	1.1	62.4	3.0	612	0.17	1:5
**4**	2.8	14.0	1.1	79.2	3.0	326	0.17	1:5
**5**	21.0	42.0	1.1	33.0	3.0	1112	0.33	1:2
**6**	14.0	28.0	1.1	54.0	3.0	756	0.33	1:2
**7**	11.2	22.4	1.1	62.4	3.0	612	0.33	1:2
**8**	5.6	11.2	1.1	79.2	3.0	326	0.33	1:2
**9**	31.5	31.5	1.1	33.0	3.0	1112	0.50	1:1
**10**	21.0	21.0	1.1	54.0	3.0	756	0.50	1:1
**11**	16.8	16.8	1.1	62.4	3.0	612	0.50	1:1
**12**	8.4	8.4	1.1	79.2	3.0	326	0.50	1:1
**13**	42.0	21.0	1.1	33.0	3.0	1112	0.67	2:1
**14**	28.0	14.0	1.1	54.0	3.0	756	0.67	2:1
**15**	22.4	11.2	1.1	62.4	3.0	612	0.67	2:1
**16**	11.2	5.6	1.1	79.2	3.0	326	0.67	2:1
**17**	52.5	10.5	1.1	33.0	3.0	1112	0.83	5:1
**18**	35.0	7.0	1.1	54.0	3.0	756	0.83	5:1
**19**	28.0	5.6	1.1	62.4	3.0	612	0.83	5:1
**20**	14.0	2.8	1.1	79.2	3.0	326	0.83	5:1

See Table S1 for information about the specific ingredients for each diet.

### 2.3 Bacterial challenge

*Xenorhabdus nematophila* is a highly pathogenic Gram-negative bacterium. In the wild, this species relies on the entomopathogenic nematode *Steinernema carpocapsae*, which vectors *X. nematophila,* to gain access to an insect host, where it rapidly multiplies, generally causing death within 24–48 h (Georgis et al., 2006; Herbert and Goodrich-Blair, 2007). However, in the lab we can circumvent the requirement for the nematode by injecting *X. nematophila* directly into the insect haemocoel (Herbert and Goodrich-Blair, 2007).

**Experiment 1:** Within 24 h of moulting to the 6th instar, 400 larvae were divided into 20 groups (n = 20 per group), placed individually into 90 mm diameter Petri dishes and provided with ∼1.5 g of one of the 20 chemically-defined diets (Table 1). Within each diet, 10 larvae were allocated to the control group and 10 were assigned to the bacteria-challenged group. Following 24 h feeding on the assigned diets (at time, t = 0), 200 larvae were handled then replaced on their diet (control) whilst 200 larvae were injected with 5 μl of a heat killed LD50 dose of *X. nematophila* (averaging 1272 *X. nematophila* cells per ml nutrient broth) using a microinjector (Pump 11 Elite Nanomite) fitted with a Hamilton syringe (gauge = 0.5 mm). The syringe was sterilised in ethanol prior to use and the challenge was applied to the left anterior proleg. Every 24 h up to 72 h (i.e. 48 h post infection), larvae were transferred individually to clean 90 mm Petri dishes containing 1.5–1.8 g of their assigned chemically-defined diet. 96 h after moulting into L6, the larvae had either pupated or were placed on semi-artificial diet until death or pupation. The amount of food eaten each day was determined by weighing the wet mass of the chemically-defined diet provided each day to the caterpillars, as well as weighing uneaten control diets each day (3 control diets per diet). The uneaten diet and control diet were then dried to a constant mass (for approx. 72 h), allowing the consumption per larva to be estimated.

**Experiment 2**: The set up for this experiment was identical to Experiment 1, except that each of the 400 larvae was injected with 5 μl of either a heat-killed (control) or live LD50 dose of *X. nematophila* (averaging 1272 *X. nematophila* cells per ml nutrient broth).

### 2.4 Hemolymph sampling

Following challenge, hemolymph samples were obtained from all caterpillars at 20 h post infection. Hemolymph samples were obtained by piercing the cuticle next to the first proleg near the head with a sterile needle and allowing released hemolymph to bleed directly into an Eppendorf tube. Immediately following hemolymph sampling, 30 μl of fresh hemolymph was added to a sterile ice-cooled Eppendorf containing 350 μl of lysis buffer (RLT + Beta mercaptoethanol – 100:1) for later RNA extraction and qPCR analysis (Expts 1 and 2). The remainder of the hemolymph extracted was stored in a separate Eppendorf for further immune assays (Expt 2 only). All hemolymph samples were stored at −80 °C prior to processing.

### 2.5 Gene expression (Expts 1 and 2)

RNA was extracted from hemolymph samples using Qiagen RNeasy mini kit following the manufacturers instructions with a final elution volume of 40 μl. Extracts were quantified using the Nanodrop 2000 and diluted to 0.5 μg/μl for cDNA synthesis. Prior to cDNA synthesis a genomic DNA elimination step was carried out by combining 12 μl RNA (0.5 μg total RNA) plus 2 μl DNA wipeout solution and incubating at 42 °C for 2 min. cDNA synthesis was carried out using Qiagen Quantitect Reverse Transcription kit in a final reaction volume of 20 μl following the manufacturer's instructions, cDNA synthesis was carried out for 30 min at 42 °C followed by 3 min incubation at 95 °C and stored at −20 °C. cDNA was diluted 1:5 for use as a qPCR template.

Primers and probes were synthesised by Primer Design and qPCR was performed in a reaction volume of 10 μl with 1x Taqman FAST Universal PCR Master mix (Thermo Fisher), 0.25 μM of each primer, 0.3 μM probe and 2 μl of a 1:5 dilution of cDNA. qPCR was carried on the ABI 7500 FAST, cycling parameters included an initial denaturation at 95 °C for 20 s followed by 40 cycles of denaturation at 95 °C, 3 s and annealing at 60 °C for 30 s. All PCRs were run in duplicate (see Table 2).

**Table 2:** Primer and probe sequences used for the qPCR analysis of immune gene expression.

Gene	Primers (5′–3′)	Probe (5′–3′)	Amplicon sizes (bp)	Efficiencies
Toll	FOR: AATGCTCGTGTTATCATGATCAAA REV: CGTGATCGTAGCCAGCGTTT	VIC-CTGGACCACCACTAACGTCGTCGATTG-TAMRA	76	93.8%
PPO	FOR: GCTGTGTTGCCGCAGAATG REV: AAATCCGTGGCGGTGTAGTC	VIC-CCGCGTATCCCGATCATCATCCC-TAMRA	67	97.4%
Lysozyme	FOR: TGTGCACAAATGCTGTTGGA REV:CGAACTTGTGACGTTTGTAGATCTTC	VIC-ACATCACCCTAGCTTCTCAGTGCGCC-TAMRA	76	96.6%
Relish	FOR: TCAACATAACAACACGGAGGAA REV: ATCAGGTACTAGGCAACTCATATC	6FAM -CCCACAAATTACTTGAAGATGAACAGGACCC-TAMRA	82	95.3%
Moricin	FOR: GGCGCAGCGATTGGTAAA REV:GGTTTGAAGAAGGAATAGACATCATG	VIC-TCTCCGGGCGATTAACATAGCCAGC- TAMRA	77	91.4%
EF1	FOR: TCAAGAACATGATCACTGGAACCT REV: CCAGCGGCGACAATGAG	6FAM -CCAGGCCGATTGCGCCGT-TAMRA	94	94.0%
Arylphorin	FOR: CGTCAGATGCAGTCTTTAAGATCTTC REV: TGCACGAACCAGTCCAGTTC	VIC-AATACCACGCCAATGGCTATCCGGTT-TAMRA	112	96.7%
Armadillo	FOR: TGCACCAGCTGTCCAAGAAG REV: AAAGCGGCAACCATTTGC	6FAM-AAGCTTCTCGCCATGCTATTATGAACTCGC-TAMRA	70	92.8%
Tubulin	FOR: CGTGGAGCCCTACAACTCTATCC REV: GCCTCGTTGTCGACCATGA	6FAM-ACCACCCACACCACCCTTGAGCAC-TAMRA	81	100%

We selected five immune genes, three from the Toll immune pathway: Toll, Prophenoloxidae (PPO), which is the precursor of the phenoloxidase enzyme (PO), responsible for production of melanin during the encapsulation response, and lysozyme, which produces the antimicrobial lysozyme enzyme, active against Gram positive bacteria. We also selected two genes from the IMD immune pathway, Moricin, which produces the AMP Moricin, active against Gram positive and negative bacteria, and Relish, which activates transcription of AMP genes (Ligoxygakis, 2013; Wiesner and Vilcinskas, 2010). We also selected three non-immune genes, Tubulin, a component of the cytoskeleton responsible for organelle and chromosomal movement. Armadillo (b-catenin), which facilitates protein-protein interactions and EF1, an elongation factor that facilitates protein synthesis. These genes were selected, due to robust amplification, from a pool of potential endogenous controls that were tested in pilot studies. We also selected Arylphorin, which is primarily characterised as a storage protein (Telfer and Kunkel, 1991), however, it is up-regulated in response to bacterial infection and also in response to non-pathogenic bacteria in the diet of *Trichoplusia ni* caterpillars (Freitak et al., 2007) and so we did not have an *a priori* expectation as to its behaviour in this species.

### 2.6 Lysozyme assays (Expt 2 only)

Bacterial agar plates were used to determine lytic activity. These were made by mixing 1.5% water agar and 1.5% freeze-dried *Micrococcus luteus* (Merck: M3770) in potassium phosphate buffer, in a 2:1 ratio. 10 ml plates of the resulting solution were poured and 2 mm diameter holes punched in each plate. Each hole was filled with 1 ml of ethanol saturated with phenylthiourea (PTU) in order to prevent melanisation of the samples. The ethanol evaporates, leaving the PTU in the hole. Following defrosting and vortexing of the stored hemolymph, each well was the filled with 1 μl of hemolymph, with two technical replicates per sample. The plates were incubated at 30°C for 24 h, and the clear zones around the samples were measured using digital callipers. Lytic activity (mg/ml) was then calculated from a serial dilution of a hen egg white lysozyme (Merck: 62971; Standard series 0.01, 0.05, 0.1, 0.5, 1 and 2 mg per ml in water).

### 2.7 Phenoloxidase assays (Expt 2 only)

Following defrosting of the hemolymph samples, 10 μl of hemolymph was added to 450 μl of NaCac buffer (1.6 g NaCac and 0.556 g CaCl_2_ l^−1^ sterile distilled water). The solution was then split into two Eppendorfs (each containing 225 μl), in order to carry out assays for both proPO and PO activity. To one Eppendorf, 25 μl of NaCac buffer was added (PO assay), and to the other, 25 μl of 20 mg per ml chymotrypsin in NaCac buffer was added (proPO activated). The samples were vortexed and incubated at 25 °C for 1 h. 90 μl of each solution was placed in a well of a 96-well microplate with 90 μl of 10 mM dopamine as a substrate. Two technical replicates were carried out per sample. Readings were taken every 15 s for 10 min at 490 nm and 25 °C using a Tecan infinite m200pro plate reader with Magellan software (V7.2). This range accounted for the linear stage of the reaction. The maximum rate of reaction was then used as an approximation of PO and proPO level. While many researchers still use L-dopa as a substrate for PO reactions, for insect POs, dopamine is the preferred substrate over L-dopa, it is the natural substrate for insects, it is more soluble than L-dopa and unlike L-dopa, is not subject to spontaneous darkening (Sugumaran, 2002).

## 3 Statistical analyses

### 3.1 Gene expression and food consumption

All statistical analyses were conducted using the *R* statistical package version 3.2.2 (R Core Team, 2018). Gene expression data were normalised using NORMA-Gene (Heckmann et al., 2011), a data driven approach that normalises gene expression relative to other genes in the dataset rather than to specifically identified reference genes. It is particularly suited to data sets with limited numbers of assayed genes. Normalised gene expression data were then standardised using the mean (μ) and standard deviation (σ) of each trait (Z = (X- μ) ⁄ σ) prior to analysis. The two experiments, run at different times, had only one treatment in common, (1 – handled vs heat-killed bacteria, 2- heat-killed vs live bacteria). For ease of interpretation, we wanted to analyse both experiments in a single model. To test the validity of this approach, we first compared the gene expression data and the data for the total amount of food consumed across both experiments for the heat-killed treatment only. There was no significant difference between any of the measures across experiments, with the exception of the total amount of food eaten, and expression of the Moricin gene. Therefore, all data were analysed in a single model, with the exception of those two response variables, where data from the two experiments were analysed separately.

Data were analysed for each gene separately using linear mixed-effects models in the packages *lme4* (Bates et al., 2015) and *lmerTest* (Kuznetsova et al., 2017). For each gene, the plate that the samples were run on was included as a random effect. A comparison was made of 90 candidate models for each gene, which comprised 30 models covering different combinations of dietary attributes (Table 3), either alone, with bacterial treatments added or with bacterial treatment interacting with the dietary traits. AIC values were corrected for finite sample sizes (AIC_c_) to establish the most parsimonious models including likely nutritional attributes driving the observed data. AIC_c_ values and *Akaike weights* were estimated using the *MuMin* package (Bartoń, 2018). The relative weight of evidence in favour of one model over another (evidence ratio) is determined by dividing the *Akaike weight* of one model by another (Burnham and Anderson, 2003). In each case, the residuals from the best model were visually inspected for deviations from normality. Gene expression for Lysozyme, Arylophorin, PPO, EF1 and Tubulin were Tukey transformed prior to reanalysis using the package *rcompanion* (Mangiafico, 2017). For visualisation of the effects of the immune challenge treatment and diet on gene expression (Fig. 2, Fig. 3, Fig. 4, Fig. 5), residuals from the null model, containing just the random plate effect (Model 1, Table 3), were plotted as thin plate splines using the package *fields* (Nychka et al., 2017). Food consumption data were analysed in the same way as the gene expression data, except only the first 20 models were used to explain consumption (Table 3).

**Table 3:** Terms included in each of the basic models describing different attributes of the diet or the amount of protein or carbohydrate consumed.

Model	Terms included in model														
	P		C	P^2^	C^2^	Co		R	Co^2^	R^2^	Pe		Ce	Pe^2^	Ce^2^
1															
2	X														
3	X			X											
4			X												
5			X		X										
6	X		X												
7	X		X	X											
8	X		X		X										
9	X		X	X	X										
10	X	*	X												
11	X	*	X	X	X										
12						X									
13						X			X						
14								X							
15								X		X					
16						X		X							
17						X		X	X						
18						X		X		X					
19						X	*	X							
20						X	*	X	X	X					
21											X				
22											X			X	
23													X		
24													X		X
25											X			X	
26											X			X	X
27											X		X		X
28											X		X	X	X
29											X	*	X		
30											X	*	X	X	X

The table shows the terms included in each of the 30 basic models covering the different dietary attributes. These models were also run including treatment as an additive or interactive effect, giving 90 models in total. **P** (protein) = grams of protein offered, **C** (carbohydrate) = grams of carbohydrate offered, **Co** (concentration) = percentage of the diet that comprises digestible nutrients (17%, 34%, 42%, 63%), **R** (ratio) = percentage of protein in the digestible component of the diet (17%, 50% or 83%); **Pe** (protein eaten) = grams of protein eaten, **Ce** (carbohydrate eaten) = grams of carbohydrate eaten. For Pe and Ce this was calculated over the first 48 h. Asterisks indicate interactions between terms (e.g. Models 10 and 11 include the interaction between protein and carbohydrate offered). Each of variables was also included as a squared term (e.g. **P**^**2**^). These 30 models were modified by including additive or interactive effects of treatment (base 90 models for all analyses). For the physiological traits and survival, the base 90 models were modified with the additive or interactive effects of expression of the relevant genes (180 models).

**Fig. 1:**
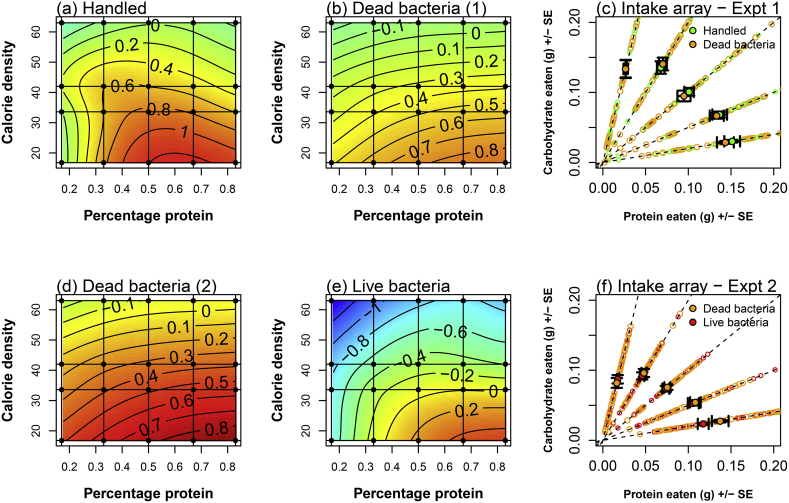
The total amount of food eaten by caterpillars that were either (a) handled or (b) injected with dead bacteria (Experiment 1) or (d) injected with dead bacteria versus (e) live bacteria (Experiment 2). Blue colours indicate low consumption and red colours high consumption. Colours are standardised within each experiment and are not comparable across experiments. Numbers on the contour lines indicate z values for consumption. Intake arrays indicating total consumption across the diets are shown separately for (c) experiment 1 and (f) experiment 2. (For interpretation of the references to colour in this figure legend, the reader is referred to the Web version of this article.)

**Fig. 2:**
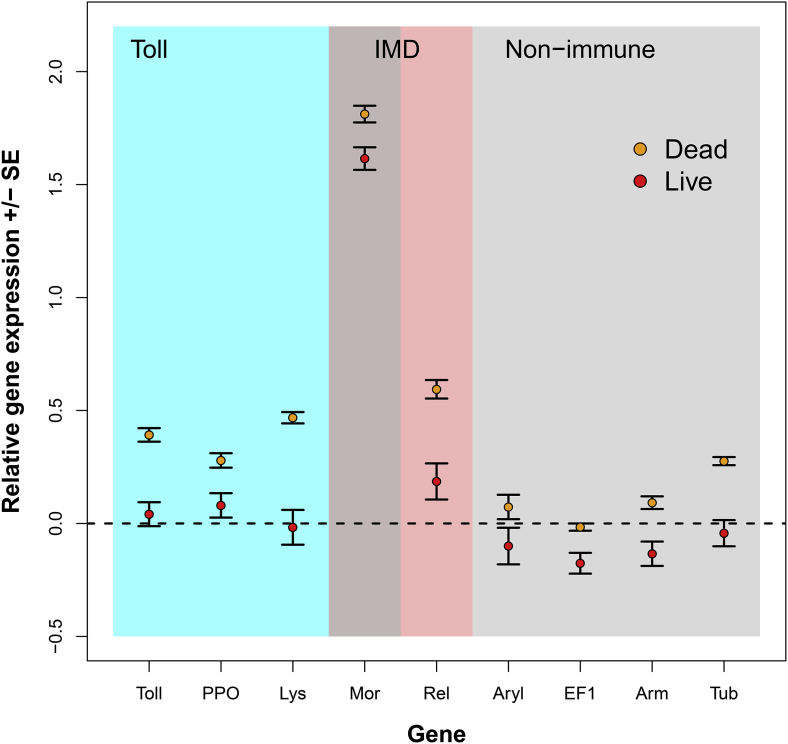
Mean gene expression (+/− SE) for each of the immune genes and non-immune genes in response to immune challenge treatment, relative to the ‘handled’ controls. Genes are grouped by immune pathway Toll (blue zone: Toll, PPO, Lysozyme and Moricin), IMD (pink zone: Moricin and Relish) or classified as non-immune genes (grey zone; Arylophorin, EF1, Armadillo and Tubulin). The black dashed line represents gene expression in the handled group. Residuals from the model accounting for the random effects of ‘plate’ are plotted against treatment. All models were re-run on untransformed data for ease of visualisation. (For interpretation of the references to colour in this figure legend, the reader is referred to the Web version of this article.)

**Fig. 3:**
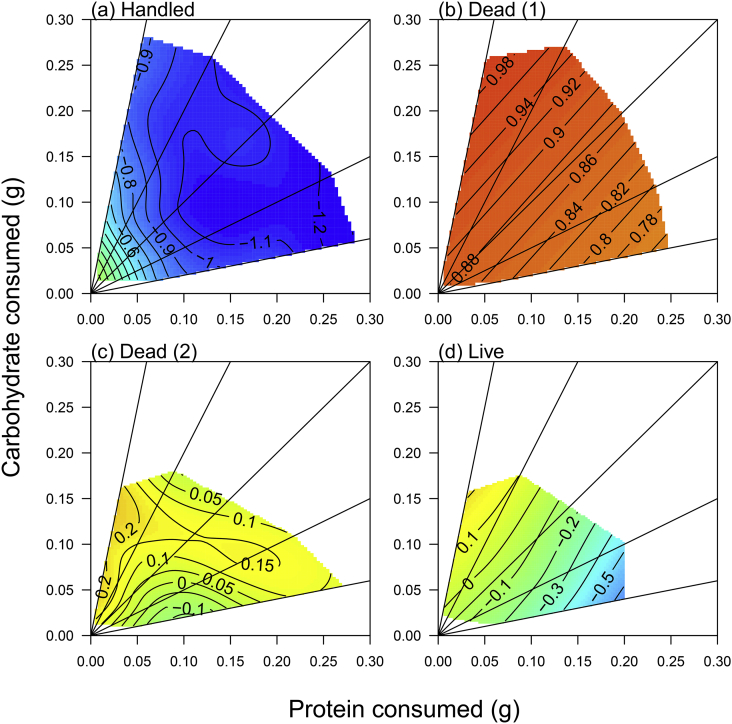
Variation in Moricin expression across diets in hemolymph of caterpillars subject to different immune challenge treatments, (a) handled only, (b) injected with dead bacteria (Expt 1), (c) injected with dead bacteria (Expt 2) and (d) injected with live bacteria. Blue colours indicate low gene expression and red colours high gene expression. Colours are standardised within each experiment and are not comparable across experiments. (For interpretation of the references to colour in this figure legend, the reader is referred to the Web version of this article.)

**Fig. 4:**
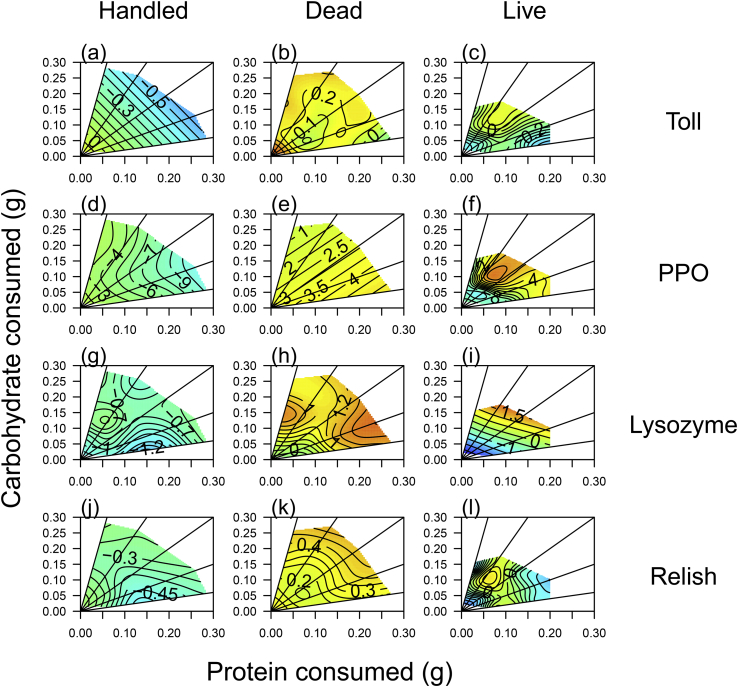
Variation in immune gene expression across diets in hemolymph of caterpillars subject to different immune challenge treatments, (a–c) Toll, (d–f) PPO, (g–i) Lysozyme and (j–l) Relish. All figures in the first column are for the handled treatment, column 2 includes those injected with dead bacteria and column 3, those injected with live bacteria. Blue colours indicate low gene expression and red colours high gene expression. (For interpretation of the references to colour in this figure legend, the reader is referred to the Web version of this article.)

**Fig. 5:**
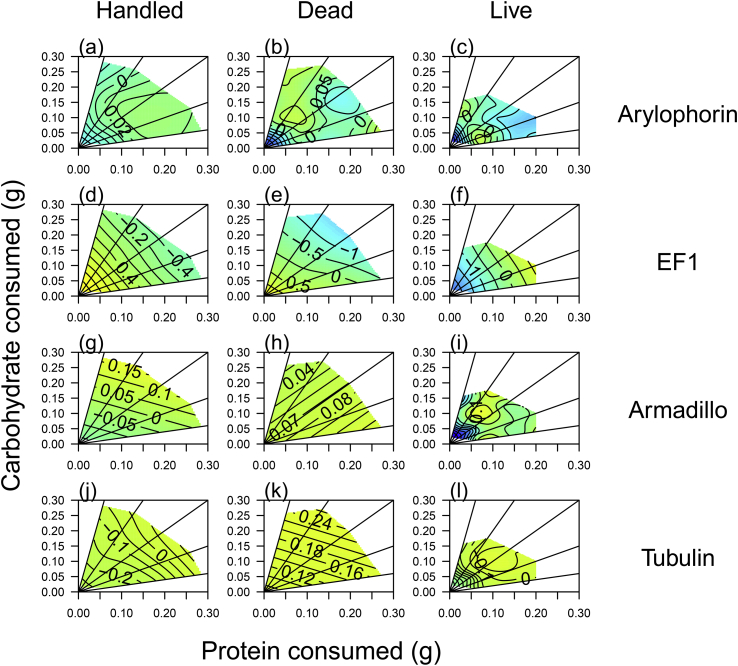
Variation in non-immune gene expression across diets in hemolymph of caterpillars subject to different immune challenge treatments, (a–c) Arylophorin, (d–f) EF1, (g–i) Armadillo and (j–l) Tubulin. All figures in the first column are for the handled treatment, column 2 includes those injected with dead bacteria and column 3, those injected with live bacteria. Blue colours indicate low gene expression and red colours high gene expression. (For interpretation of the references to colour in this figure legend, the reader is referred to the Web version of this article.)

### 3.2 Physiological immune responses

The same approach was taken for the physiological immune measurements, lysozyme, PPO and PO activity, except for these variables, standard linear fixed effects models were run as data were collected in a single experiment. The same set of 90 models as described above were fitted, with the addition of 180 extra models that included the additive and interactive effects of the expression of the relevant gene, after correction for the plate to plate variation (residuals from the null model containing the random effect of plate only) – the lysozyme gene for lysozyme activity and the PPO gene for PPO and PO activity.

### 3.3 Survival

Time to death was analysed for experiment 2, where larvae were injected with dead or live bacteria only. Data were analysed using Cox's proportional hazard models in the package *survival* (Therneau, 2015). The same sets of models as described above were fitted (Table 3), with the addition of 180 extra models that included the additive and interactive effects of Moricin gene expression, after correction for the plate to plate variation (residuals from the null model containing the random effect of plate only). For visualisation of the effects of the immune challenge treatment on survival (dead vs live bacteria), predicted curves for low and high levels of Moricin gene expression were generated using the package *Survminer* (Kassambara and Kosinski, 2018) using ggplot2 (Wickham, 2016). The effects of diet on time to death were plotted as thin plate splines using the package *fields* (Nychka et al., 2017).

## 4 Results

### 4.1 How does consumption vary across diets and bacterial challenge treatments?

The total amount of food consumed varied across the diets. For experiment 1, comparing handled caterpillars versus those injected with heat-killed bacteria, the best model predicting consumption was model   20 (Co*R + Co^2^+R^2^), but this was indistinguishable from the same model that included the additive effects of treatment (Treatment +    Co*R + Co^2^+R^2^, delta = 1.34).

For experiment 2, comparing dead and live bacterial injections, the best model predicting consumption was model 20 (Co*R + Co^2^+R^2^), but as for the handled versus dead treatments in experiment 1, this model was indistinguishable from the same model that included the additive effects of treatment (Treatment + Co*R + Co^2^+R^2^, delta = 0.51).

For all treatment groups, it can be seen that consumption tended to increase as the calorie density of the diet decreased (Fig. 1a,b,d,e), suggesting that food dilution constrained caterpillars from being able to take in sufficient nutrients, as expected, and that on the more calorie-dense diets caterpillars over-consumed nutrients. However, this increase in total consumption was more extreme on the high-protein than on the low-protein diets, suggesting that caterpillars were willing to overeat protein to gain limiting carbohydrates.

Overall consumption tended to decrease with treatment - dead-bacteria treated caterpillars ate less than handled, and live-bacteria treated caterpillars ate less than dead-bacteria treated (Fig. 1a vs b and d vs e). However, inspection of the intake arrays (Fig. 1c,e), suggests that consumption was most reduced in both dead and live bacteria treatments on the highest protein diets.

### 4.2 How does immune gene expression vary across diets and bacterial challenge treatments?

For the immune genes (Toll, PPO, Lysozyme, Moricin and Relish), injection with dead bacteria resulted in up-regulation of gene expression relative to handled caterpillars (Fig. 2). In contrast, injection with live bacteria either did not up-regulate gene expression relative to controls (Toll, PPO and Lysozyme), or did not up-regulate it as strongly (Moricin and Relish) (Fig. 2). For the non-immune genes (Arylophorin, EF1, Armadillo and Tubulin), the variation in expression levels was lower; for Arylophorin, EF1 and Armadillo, live bacteria triggered the down-regulation of gene expression relative to handled caterpillars, whilst there was no effect for Tubulin (Fig. 2). For Arylophorin, Armadillo and Tubulin, injection with dead bacteria up-regulated gene expression relative to handled caterpillars but there was no effect for EF1 (Fig. 2). The best supported model for every gene tested was model 30, with the bacterial treatment interacting with the amount of protein and carbohydrate eaten (Treatment*(Pe*Ce + Pe^2^+Ce^2^)). However, although the fit of these models was generally good (r^2^ > 0.26–0.86), with the exception of Moricin, the amount of variation explained by the fixed part of the model was very low (r^2^ < 0.12; Table 4; Fig. 3, Fig. 4, Fig. 5). This means that the majority of the variation in gene expression was caused by variation across plates. For Moricin, when comparing the handled and dead treatments, 74% of the variation explained by the model was explained by the fixed terms due to the massive up-regulation of Moricin in the bacteria-injected larvae relative to handled controls (Fig. 2, Fig. 3a,b). The difference between the dead and live treatment groups was much smaller and comparable to the other immune genes (Table 4, Fig. 3c and d).

**Table 4:** The best model selected by AICc to explain variation in gene expression across the diet and bacterial treatments.

Gene	Best Model	R^2^ fixed	R^2^ both
Toll	Treat*30	0.059	0.717
PPO	Treat*30	0.035	0.715
Lysozyme	Treat*30	0.104	0.736
Relish	Treat*30	0.120	0.378
Moricin (1)	Treat*30	0.741	0.741
Moricin (2)	Treat*30	0.097	0.264
Arylphorin	Treat*30	0.089	0.275
EF1	Treat*30	0.023	0.862
Armadillo	Treat*30	0.034	0.696
Tubulin	Treat*30	0.040	0.855

Variation in the expression of all of the genes was explained by main and interactive effects of the amount of protein and carbohydrate eaten, and in interaction with the bacterial treatment, suggesting that the response to diet for each gene differed across treatments. A visualisation of these response surfaces (Fig. 3, Fig. 4, Fig. 5) shows that, for the immune genes, whilst there is general up-regulation between handled and dead bacterial challenges, the response surfaces are fairly flat, i.e. diet does not explain much variation in gene expression. However, for the live challenge, expression tends to peak at moderate protein but high carbohydrate intake, which corresponds to the highest intakes on the 33% protein diet for Toll, PPO, Lysozyme and Relish, and on the 17% protein diet for Moricin (Fig. 3, Fig. 4). In contrast, the non-immune genes (Arylphorin, EF1, Armadillo and Tubulin), show a consistently weak response to the dietary manipulation, with much flatter surfaces on average than those shown by the immune genes (compare Fig. 4 with Fig. 5).

### 4.3 Does immune gene expression predict physiological immune responses?

For the Lysozyme and PPO genes, we simultaneously measured functional lytic and PPO (and PO) activity in the hemolymph, allowing us to determine how well gene expression predicts the functional immune response. We had lytic and PO data only for Experiment 2, where larvae were challenged with live or dead bacteria.

For PPO activity, AICc could not discriminate between several of the diet models, with seven being equally well supported (delta <2; Table 5). Of these models, the top six contained protein and protein squared with additive or interactive effects of bacterial treatment or gene expression (Table 5). For the models that included treatment, the estimates show that PPO activity increased with live bacterial infection. For PO activity, AICc could not discriminate between 11 different models (delta <2; Table 6). However, the top three models were the same as for PPO, with protein plus protein squared with additive or interactive effects of PPO gene expression. Only two of the models contained treatment effects and both in interaction with diet components. For lytic activity in the hemolymph, three models were equally well supported, all of which contained Lysozyme gene expression interacting with dietary components, which were either protein and protein squared, as for PO and PPO, or the P:C ratio (Table 7); none of the models contained treatment, suggesting that lysozyme activity is up-regulated in response to the presence of bacteria and not whether they are alive or dead. As for gene expression, the overall explanatory power of the models was quite low, (r^2^ < 0.12; Table 5, Table 6, Table 7).

**Table 5:** The best models selected by AICc to explain variation in PPO activity in the hemolymph. GE represents gene expression for the PPO gene. Treat represents the immune challenge treatment.

Model	df	Log Likelihood	AICc	delta	weight	R^2^
3	4	−432.120	872.4	0.00	0.078	0.093
GE+3	5	−431.259	872.7	0.34	0.066	0.098
GE*3	7	−429.321	873.0	0.64	0.057	0.109
Treat+3	5	−431.737	873.7	1.30	0.041	0.095
Treat + GE*3	8	−428.617	873.7	1.34	0.040	0.113
Treat + GE+3	6	−430.716	873.7	1.34	0.040	0.101
7	5	−431.938	874.1	1.70	0.033	0.094

**Table 6:** The best models selected by AICc to explain variation in PO activity in the hemolymph. GE represents gene expression for the PPO gene. Treat represents the immune challenge treatment.

Model	df	Log Likelihood	AICc	delta	weight	R^2^
GE*3	7	−425.954	866.3	0.00	0.062	0.092
GE+3	5	−428.058	866.3	0.04	0.061	0.080
3	4	−429.363	866.9	0.58	0.047	0.072
GE+16	5	−428.350	866.9	0.62	0.046	0.078
GE+9	7	−426.378	867.1	0.85	0.041	0.090
16	4	−429.513	867.2	0.88	0.040	0.071
GE + Treat*17	10	−423.239	867.2	0.93	0.035	0.108
Treat*17	9	−424.471	867.5	1.26	0.033	0.100
GE+17	6	−427.775	867.8	1.55	0.029	0.081
GE+10	8	−425.777	868.0	1.75	0.026	0.093
GE+19	6	−427.887	868.0	1.77	0.026	0.081

**Table 7:** The best models selected by AICc to explain variation in lytic activity in the hemolymph. GE represents gene expression for the lysozyme gene.

Model	df	Log Likelihood	AICc	delta	weight	R^2^
GE*15	7	647.521	−1280.7	0.00	0.208	0.072
GE*18	9	649.465	−1280.3	0.34	0.176	0.080
GE*3	5	644.526	−1278.9	1.82	0.084	0.051

**Fig. 6:**
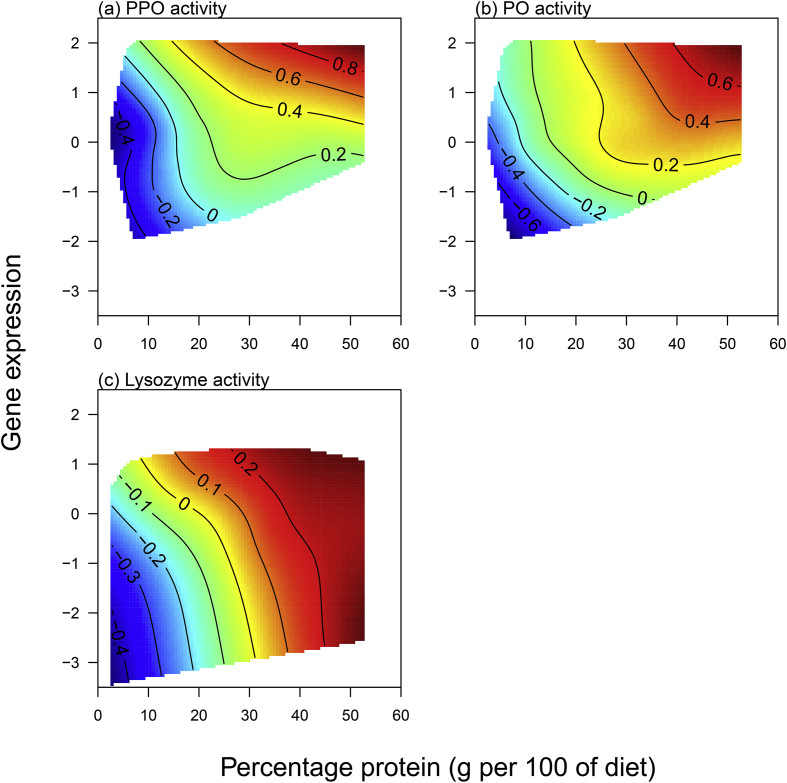
Physiological immune responses vary with the protein content of the diet and the expression of the relevant gene. (a) PPO and (b) PO activity in the hemolymph in response to PPO gene expression and (c) lysozyme activity in the hemolymph in response to lysozyme gene expression. Blue colours indicate low activity and red colours high activity. (For interpretation of the references to colour in this figure legend, the reader is referred to the Web version of this article.)

For ease of comparison, all 3 physiological immune traits were plotted against the protein content of the diet, as this model was common to all three traits, and the expression of the relevant gene, which featured in the majority of the selected models (Table 5, Table 6, Table 7). The effect of treatment was excluded as it did not feature in the majority of the models. For each trait, activity in the hemolymph tended to increase with gene expression, as we might expect, but this was strongly moderated by the protein content of the diet (Fig. 6). For PO and PPO activity, on low protein diets enzyme activity was low and there was little correspondence between gene expression and the physiological response, but as the protein content of the diet increased, this relationship became more linear (Fig. 6a and b). For lytic activity the pattern was different in that enzyme activity increased strongly with the protein and less strongly with lysozyme gene expression up to about 45% protein, thereafter there was consistently high lytic activity across all levels of gene expression (Fig. 6c).

### 4.4 Does immune gene expression predict survival?

**Fig. 7:**
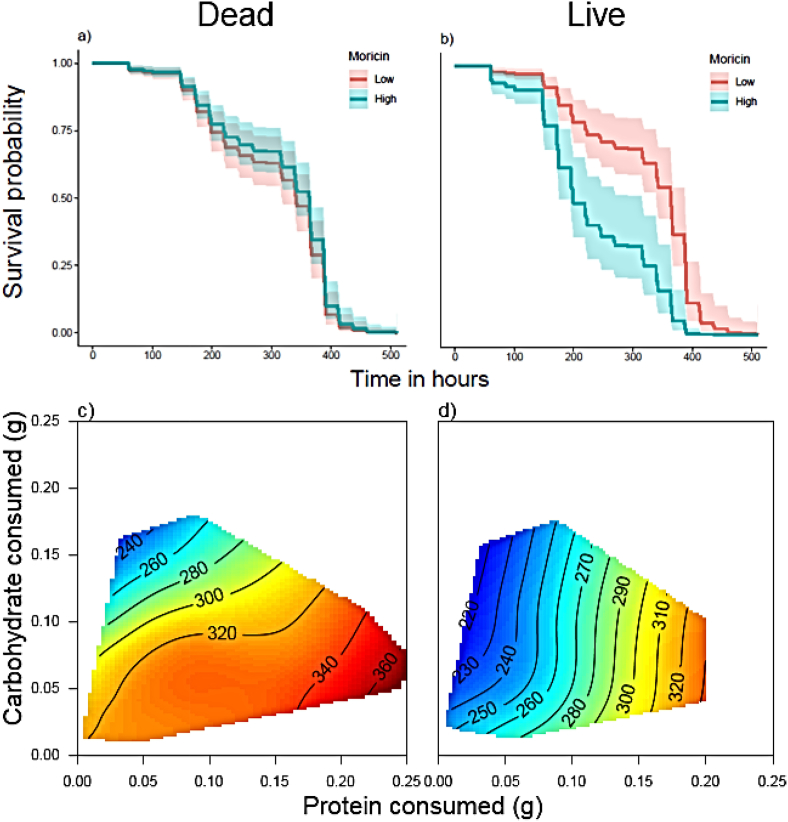
Survival for larvae injected with either dead (a,c) or live (b,d) *X. nematophila* bacteria. Predicted survival curves (a,b) are plotted for model Treat*GE*30. Protein eaten and carbohydrate eaten were set at mean values for each coefficient and Moricin gene expression was set as either low (lower quartile) or high (upper quartile). To visualise the effects of diet on survival, time to death in hours post injection (c,d) is plotted as thin plate splines against the amount of protein and carbohydrate consumed. Blue colours indicate a short time to death and red colours a slow time to death. (For interpretation of the references to colour in this figure legend, the reader is referred to the Web version of this article.)

Survival was reduced in the live bacterial treatment group relative to those injected with dead bacteria (Hazard ratios 1.25–1.31 for models without treatment interactions, Table 8), however, this effect was moderated by Moricin expression (Fig. 7 a,b). In the dead-bacteria treatment group, Moricin did not explain time to death, but in the live-bacteria treatment group, larvae with high levels of Moricin expression had an increased risk of death relative to those with low expression (Fig. 7 a,b; Hazard ratios 1.20–1.24 for models without GE interactions, Table 8). Of the top 5 models, 4 included the additive and interactive effects of protein and carbohydrate eaten as well as their squared terms (Table 8). To visualise the effects of diet on survival we plotted thin-plate splines for time to death against the amount of protein and carbohydrate consumed. The patterns differed between dead and live bacterial treatments. Time to death was overall shorter in the live treatment (note the shift of colour towards orange and blue). However, whilst time to death was affected by both protein and carbohydrate consumption in the dead treatment, with peak survival on high protein/low carbohydrate, in the live treatment, time to death appeared to be solely explained by protein consumption (note the near-vertical contours). Low-protein diets resulted in the most rapid deaths and high-protein diets extended the time to death.

**Table 8:** The best models selected by AICc to explain variation in survival after bacterial (dead or live) injection. GE represents gene expression for the Moricin gene.

Model	df	Log Likelihood	AICc	delta	weight	R^2^
Treat + GE*30	12	−1662.917	3350.8	0.00	0.139	0.127
Treat*GE*30	23	−1650.823	3351.1	0.30	0.120	0.186
Treat + GE+30	7	−1668.715	3351.8	0.99	0.085	0.098
Treat + GE+20	7	−1668.864	3352.1	1.29	0.073	0.097
GE*30	11	−1664.796	3352.4	1.61	0.062	0.118

## 5 Discussion

Previous work has shown that immune responses can be strongly affected by the amount and/or balance of nutrients in the diet e.g. (Fernandes et al., 1976; Ingram et al., 1995; Kristan, 2008; Le Couteur et al., 2015; Lee et al., 2006; Nayak et al., 2009; Povey et al., 2009; Ritz and Gardner, 2006; Wallace et al., 1999; White et al., 2017). However, most of these studies covered only a relatively small region of nutrient space (Fernandes et al., 1976; Ingram et al., 1995; Lee et al., 2006; Nayak et al., 2009; Povey et al., 2009; Ritz and Gardner, 2006; White et al., 2017) and/or only tested innate responses (e.g.(Fernandes et al., 1976; Ingram et al., 1995; Le Couteur et al., 2015; Lee et al., 2006; Nayak et al., 2009; Povey et al., 2009; White et al., 2017) or the response to an artificial pathogen or immune stimulant (Cotter et al., 2011b). Here we addressed this gap by looking at both gene expression, functional immune responses and survival after both dead and live pathogen challenges over a broad region of nutrient space. Our major findings are that whilst functional immune responses (PPO, PO and lytic activity in the hemolymph) change as expected in response to the dietary manipulation, showing a clear elevation as the protein content of the diet increases, gene expression is much less predictable (Fig. 3, Fig. 4). Despite this, expression of the PPO and Lysozyme genes did predict PPO/PO and Lysozyme activity in the hemolymph, but this relationship was strongly dependent on the amount of protein in the diet (Fig. 6), suggesting that using immune gene expression as an indicator of the efficacy of the immune response may be reliable only under specific dietary conditions. Furthermore, expression of the most responsive gene to infection (Moricin) strongly modulated survival, with high levels of expression resulting in reduced survival after bacterial infection, suggesting that expression is a marker of bacterial load or ‘sickness’ as opposed to an indication of a robust immune response.

Our dietary manipulation was successful in inducing caterpillars to consume over a large region of nutrient space, allowing us to independently assess the effects of macronutrient composition and the calorie content of the diet on immunity. There was evidence for compensatory feeding; caterpillars did not consume the same amount of every diet. As expected, caterpillars ate more as the calorie density of the food decreased (Fig. 1), but this varied across diets, such that consumption was highest on the high protein diets, suggesting that caterpillars were willing to over-eat protein to gain limiting carbohydrates. However, as has been found in previous studies (Adamo, 1998; Adamo et al., 2007; Exton, 1997; Lennie, 1999; Povey et al., 2014), we found some evidence for illness-induced anorexia. Caterpillars injected with live *X. nematophila* showed suppressed food consumption across all diets (Fig. 1e – note the shift of colours towards oranges and blues). Interestingly, injection with dead *X. nematophila* did not induce this response, which suggests that it is not the triggering of an immune response that causes this change in consumption, but the presence of an actively replicating pathogen..

In insects, two major pathways are triggered in response to microbial infection; typically, genes in the Toll pathway respond to infection by fungi and Gram-positive bacteria, whilst genes in the IMD pathway respond to Gram-negative bacteria (Broderick et al., 2009). Moricin and Lysozyme are triggered by Toll in Lepidoptera (e.g. (Zhong et al., 2016), but Moricin has also been shown to respond to Gram-negative bacteria and so may also be triggered by IMD (Hara and Yamakawa, 1995). Of the 5 immune genes we tested, only the IMD genes, Moricin and Relish, were significantly up-regulated in response to infection with both dead and live bacteria. For the Toll genes (Toll, PPO and Lysozyme), gene expression was up-regulated by dead bacteria but not by live bacteria (Fig. 2). However, even for Moricin and Relish, up-regulation was much stronger in response to dead than live bacteria. This may reflect a general down-regulation of gene expression during an active infection, as the non-immune genes typically show reduced gene expression in response to the live infection compared to the controls. This may be driven by illness-induced anorexia, with reduced consumption resulting in lower metabolic activity and consequently lower gene expression. However, there is evidence that *X. nematophila* can inhibit Cecropin, Attacin and Lysozyme gene expression (Ji and Kim, 2004; Park et al., 2007). It may be that, rather than specifically inhibiting AMP gene expression, *X. nematophila* inhibits the expression of all genes.

As Moricin was most strongly up-regulated in response to infection, we tested how its expression correlated with time to death in challenged caterpillars (dead vs live injection, Expt 2 only). Whilst Moricin expression had negligible effects on survival in the dead bacterial treatment, high levels of expression were indicative of an *increased* risk of death after live infection. Thus, high expression levels were not a good indicator of immune capacity, but rather signalled heavy bacterial loads or low tolerance. Distinguishing between these hypotheses would require data on bacterial loads at different time points after infection. Survival was also strongly affected by diet, with the longest survival times occurring on the highest protein diets after live-bacteria infection. High-protein diets have been implicated in increased survival after viral infection in this species (Lee et al., 2006) and after either bacterial or viral infection in the congener, *Spodoptera exempta* (Povey et al., 2009, 2014). However, none of these diets are associated with the highest gene expression for any immune gene, suggesting that high-protein diets may reduce the burden of infection via mechanisms other than improving the immune response.

*X. nematophila* is a Gram-negative bacterium, and is clearly triggering Moricin and Relish expression, but as Toll is only marginally up-regulated in response, it is probably the IMD pathway that is controlling this response. Another possible explanation for why live bacteria appear to trigger a down-regulation of gene expression is that our sampling protocol (20 h post-challenge) did not allow us to catch peak expression levels (note that bacterial loads tend to peak in *S. littoralis* at around 24 h). Expression of lysozyme and PPO in the Glanville fritillary butterfly was not up-regulated 24 h after injection with *M. luteus* cells (Woestmann et al., 2017), whilst in the silkworm, up-regulation of lysozyme in response to fungal infection occurred in two peaks, from 9 to 18 h, and then between 30 and 48 h (Hou et al., 2014). This may be a fungal-specific response, or it might mean that we would have seen higher gene expression had we assayed over an extended time period. It is also possible that the timing of gene expression peaks earlier after live, rather than dead bacterial injection, further studies would be required to elucidate the time course of gene expression for the different genes to be certain of this. However, as non-immune genes also appear to follow the same pattern, reduced expression in response to live vs dead bacteria, the hypothesis that infection results in down-regulation of gene expression in general is a reasonable assumption.

Arylphorin is primarily characterised as a storage protein (Telfer and Kunkel, 1991), however, it is up-regulated in response to bacterial infection and also in response to non-pathogenic bacteria in the diet of *Trichoplusia ni* caterpillars (Freitak et al., 2007). It has been shown to bind to fungal conidia in *Galleria mellonella* hemolymph, potentially working in coordination with antimicrobial peptides (Fallon et al., 2011). The lack of up-regulation here may be due to the use of a Gram-negative bacterial challenge; the up-regulation in *T. ni* was in response to a mixture of *E. coli* (G-ve) and *Micrococcus luteus* (G + ve), so it is not clear if both or just one of the bacteria caused the response. Another possibility is that Arylphorin levels are already expressed at maximal levels and cannot be further up-regulated. In *T. ni* caterpillars, Arylphorin is the most abundant protein in the hemolymph during the final instar (Kunkel et al., 1990). Its levels are known to increase throughout the final instar in *Spodoptera litura* (Yoshiga et al., 1997), and the point at which gene expression was measured here was 48–72 h into the final instar, which is shortly before pupation. The pattern of gene regulation for Arylphorin looks more like that shown by the non-immune genes, with little or no up-regulation in response to dead bacteria and down-regulation in response to live bacteria. Further studies would be required to assess the role of Arylphorin as a putative immune gene in this species.

For two of the immune genes, PPO and Lysozyme, we were able to simultaneously measure the activity of the relevant protein in the hemolymph as a measure of the functional immune response. Thus, we were able to assess how well gene expression predicts functional immune activity and whether this relationship changes with the diet. Here, we found that for each functional immune response, PPO activity, PO activity and lysozyme activity, expression of the relevant gene does predict the response, but only at certain intakes of protein (Fig. 6). For example, PPO and PO activity increase linearly with the expression of the PPO gene, but only above ∼30% dietary protein (Fig. 6). This suggests that the availability of dietary protein limits the translation of PPO mRNA into PPO protein, and the activation of PPO into PO. In contrast, the expression of the gene is not limited by protein availability, and so gene expression can be high when dietary protein is low, but it is ineffective as it does not result in a comparable functional immune response. The lytic response is also affected by dietary protein, however, in this case, the relationship between gene expression and lytic activity is consistently weak and above 45% protein maximal lytic activity is achieved at low gene expression, and increased expression does not improve the response. As for PPO, this suggests that protein limits the translation of lysozyme up to about 45% protein.

These results are not surprising when you consider the costs associated with the production of protein. It is estimated that only 10% of the energetic costs of protein production are spent on transcription; translation is much more energetically expensive and relies on the availability of amino acids to build the relevant protein (Warner, 1999). It is likely, therefore, that whilst transcription of immune genes might still be up-regulated in response to infection under low protein conditions, the translation of the protein might be reduced, impairing the correlation between mRNA and protein abundance. It is also possible that gene expression would be a better predictor of the functional response at different time points, if there is a lag between gene expression and protein translation. Again, this would require further investigation. However, given the much stronger relationship between the physiological immune responses and protein availability, it still seems likely that the relationship between the two will differ across diets. Our results suggest that caution should be used when interpreting gene expression as a measure of “investment” into a particular trait, or as a measure of the strength of a particular immune response. It is surprisingly common in ecological studies for gene expression to be used in this way without any attempt to correlate the expression of a gene with the production of the functional protein (Zylberberg, 2019). If dietary protein levels are limiting, then gene expression may be a poor indicator of the immune capacity of an animal. Here we have tested this just with immune genes for which we have good functional assays of the active protein, but it seems likely that this would also be true of other classes of genes, for which gene expression is routinely used as an indicator of an organism's investment.

In summary, as expected, immune challenge with a live Gram-negative bacterium up-regulated immune genes in the IMD pathway, though all immune genes were up-regulated to a certain extent by the challenge with dead bacteria. While functional immune responses (PO, PPO and Lysozyme) typically improved with the protein content of the diet, gene expression varied non-linearly with diet composition. However, the expression of PPO and Lysozyme genes predicted PPO/PO and Lysozyme activity, but only when the availability of dietary protein was not limiting, suggesting that using gene expression as an indicator of investment in a trait is unlikely to be reliable, unless its relationship with diet is known.
