# Supplementary material for: Diet modulates the relationship between immune gene expression and functional immune responses
Source: Insect Biochem Mol Biol. 2019 Jun;109:128–41. doi: 10.1016/j.ibmb.2019.04.009 (PMC6527921; doi:10.1016/j.ibmb.2019.04.009)
Supplement: Multimedia component 2 [file mmc2.docx]

| **Diet** | **Cellulose** | **Casein** | **Peptone** | **Albumen** | **Linoleic acid** | **Cholesterol** | **Sucrose** | **Wessons salts** | **Ascorbate** | **Vitamin mix** |
| --- | --- | --- | --- | --- | --- | --- | --- | --- | --- | --- |
| **1** | 33 | 6.3 | 2.1 | 2.1 | 0.55 | 0.55 | 52.5 | 2.5 | 0.275 | 0.18 |
| **2** | 54 | 4.2 | 1.4 | 1.4 | 0.55 | 0.55 | 35 | 2.5 | 0.275 | 0.18 |
| **3** | 62.4 | 3.36 | 1.12 | 1.12 | 0.55 | 0.55 | 28 | 2.5 | 0.275 | 0.18 |
| **4** | 79.2 | 1.68 | 0.56 | 0.56 | 0.55 | 0.55 | 14 | 2.5 | 0.275 | 0.18 |
| **5** | 33 | 12.6 | 4.2 | 4.2 | 0.55 | 0.55 | 42 | 2.5 | 0.275 | 0.18 |
| **6** | 54 | 8.4 | 2.8 | 2.8 | 0.55 | 0.55 | 28 | 2.5 | 0.275 | 0.18 |
| **7** | 62.4 | 6.72 | 2.24 | 2.24 | 0.55 | 0.55 | 22.4 | 2.5 | 0.275 | 0.18 |
| **8** | 79.2 | 3.36 | 1.12 | 1.12 | 0.55 | 0.55 | 11.2 | 2.5 | 0.275 | 0.18 |
| **9** | 33 | 18.9 | 6.3 | 6.3 | 0.55 | 0.55 | 31.5 | 2.5 | 0.275 | 0.18 |
| **10** | 54 | 12.6 | 4.2 | 4.2 | 0.55 | 0.55 | 21 | 2.5 | 0.275 | 0.18 |
| **11** | 62.4 | 10.08 | 3.36 | 3.36 | 0.55 | 0.55 | 16.8 | 2.5 | 0.275 | 0.18 |
| **12** | 79.2 | 5.04 | 1.68 | 1.68 | 0.55 | 0.55 | 8.4 | 2.5 | 0.275 | 0.18 |
| **13** | 33 | 25.2 | 8.4 | 8.4 | 0.55 | 0.55 | 21 | 2.5 | 0.275 | 0.18 |
| **14** | 54 | 16.8 | 5.6 | 5.6 | 0.55 | 0.55 | 14 | 2.5 | 0.275 | 0.18 |
| **15** | 62.4 | 13.44 | 4.48 | 4.48 | 0.55 | 0.55 | 11.2 | 2.5 | 0.275 | 0.18 |
| **16** | 79.2 | 6.72 | 2.24 | 2.24 | 0.55 | 0.55 | 5.6 | 2.5 | 0.275 | 0.18 |
| **17** | 33 | 31.5 | 10.5 | 10.5 | 0.55 | 0.55 | 10.5 | 2.5 | 0.275 | 0.18 |
| **18** | 54 | 21 | 7 | 7 | 0.55 | 0.55 | 7 | 2.5 | 0.275 | 0.18 |
| **19** | 62.4 | 16.8 | 5.6 | 5.6 | 0.55 | 0.55 | 5.6 | 2.5 | 0.275 | 0.18 |
| **20** | 79.2 | 8.4 | 2.8 | 2.8 | 0.55 | 0.55 | 2.8 | 2.5 | 0.275 | 0.18 |
| **Order code** | C8002  Sigma | C7078  Sigma | 70951  Sigma | A5253  Sigma | 62240  Sigma | C8503  Sigma |  | F8680  Bioserv | A596  Sigma | F8135  Bioserv |

Table S1 – diet ingredients of the 20 chemically-defined diets

.

All values are in grams for 100g of each diet. Proteins are in brown, fats in yellow and carbohydrate in orange. Vitamins and minerals (blue) were adding at a constant level of 3g per 100. Fats were dissolved in chloroform before being added to the casein and cellulose component of the diet to ensure an even distribution throughout the diet. The chloroform was allowed to evaporate overnight in a fume cupboard before the addition of the remaining ingredients. The resulting dry powders were stored in the freezer until use. Prior to use the powder was diluted 1:6 in 1% agar and allowed to set. The cellulose, fats, proteins and ascorbate were purchased from Sigma, the Wessons salts and Vitamin mix were purchased from Bioserv and the sucrose was table top sugar.
